# The role of KRAS splice variants in cancer biology

**DOI:** 10.3389/fcell.2022.1033348

**Published:** 2022-11-01

**Authors:** Cristina Nuevo-Tapioles, Mark R. Philips

**Affiliations:** Perlmutter Cancer Center, NYU School of Medicine, New York, NY, United States

**Keywords:** *KRAS*, KRAS4A, KRAS4B, alternative splicing, oncogene, oncoprotein, glycolysis

## Abstract

The three mammalian *RAS* genes (*HRAS*, *NRAS* and *KRAS*) encode four proteins that play central roles in cancer biology. Among them, *KRAS* is mutated more frequently in human cancer than any other oncogene. The pre-mRNA of *KRAS* is alternatively spliced to give rise to two products, KRAS4A and KRAS4B, which differ in the membrane targeting sequences at their respective C-termini. Notably, both KRAS4A and KRAS4B are oncogenic when *KRAS* is constitutively activated by mutation in exon 2 or 3. Whereas KRAS4B is the most studied oncoprotein, KRAS4A is understudied and until recently considered relatively unimportant. Emerging work has confirmed expression of KRAS4A in cancer and found non-overlapping functions of the splice variants. The most clearly demonstrated of these is direct regulation of hexokinase 1 by KRAS4A, suggesting that the metabolic vulnerabilities of *KRAS*-mutant tumors may be determined in part by the relative expression of the splice variants. The aim of this review is to address the most relevant characteristics and differential functions of the *KRAS* splice variants as they relate to cancer onset and progression.

## Introduction

**FIGURE 1:**
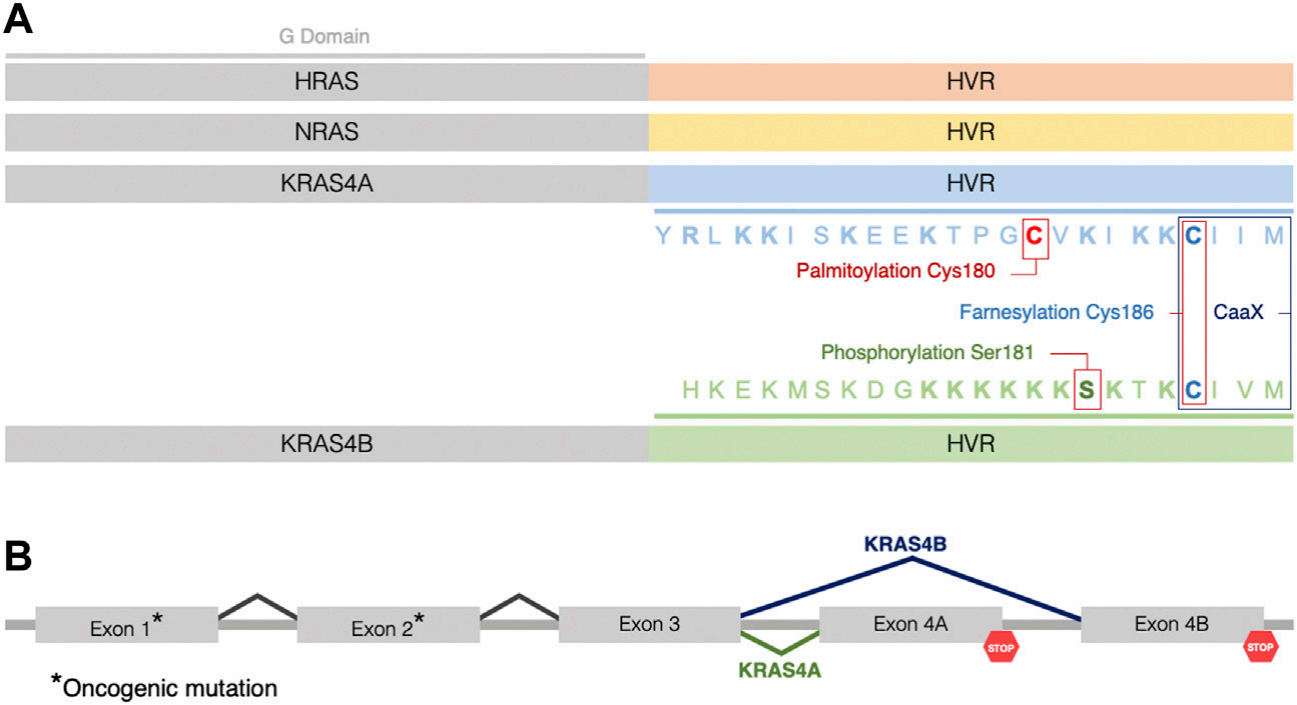
Three *RAS* genes encode four RAS proteins. **(A)** The four RAS isoforms have nearly identical GTP/GDP binding (G) domains (grey) (aa1–165) but distinct hypervariable (HVR) regions (aa166–188/189, not to scale), which direct membrane trafficking. Color coding of the HVR distinguishes each RAS isoform (HRAS in orange, NRAS in yellow, KRAS4A in blue, and KRAS4B in green). Sequence and posttranslational modifications of the HVR of KRAS4A and KRAS4B are indicated by colors highlighting the residues that are palmitoylated (red), phosphorylated (green) or farnesylated (blue). Basic residues in the HVRs of KRAS4A and KRAS4B are indicated in bold. **(B)** The two KRAS isoforms are splice variants utilizing alternative 4th exons. Oncogenic mutations occur in exons 1 or 2 (*) such that when *KRAS* is mutant, both splice variants give rise to oncogenic proteins.

The three mammalian *RAS* genes (*HRAS*, *NRAS* and *KRAS*) encode four proteins that play central roles in cancer biology (Prior et al., 2012) (Figure 1). The *RAS* genes encode small GTPases that regulate cellular pathways that signal for growth, proliferation, and differentiation. The activation of RAS proteins is determined by nucleotide binding, with the GTP-bound form assuming an active signaling conformation (Vigil et al., 2010; Simanshu et al., 2017). Missense mutations in RAS proteins lead to an altered homeostatic balance of GDP and GTP binding of RAS toward the active state, either by reducing GTP hydrolysis or by increasing the rate of GTP loading (Cox and Der, 2010; Hobbs et al., 2016). When in the GTP-bound, activated state RAS proteins signal by engaging effectors that transmit signals down several pathways (Stephen et al., 2014; Simanshu et al., 2017). Whereas more than 10 effectors have been described (Kiel et al., 2021), RAF proteins are the effectors that regulate the MAPK pathway and are the *sine qua non* of oncogenic signaling. Although there is some evidence that the different RAS proteins engage the various effectors with different efficiencies (Voice et al., 1999), all GTP-bound RAS proteins can engage all effectors *in vitro*. Nevertheless, differential subcellular localizations can drive compartmentalized signaling by selecting for distinct subsets of effectors (Chiu et al., 2002; Mor and Philips, 2006; Prior and Hancock, 2012).

*RAS* genes are the most frequently mutated oncogenes in cancer and among the *RAS* genes, *KRAS* mutations are most frequently associated with tumors (Prior et al., 2012). Interestingly, the *KRAS* transcript is alternatively spliced giving rise to two variants with alternative 4th exons, designated KRAS4A and KRAS4B (Ahearn et al., 2011) (Figure 1). These variants encode nearly identical G domains (aa1-165) that bind guanine nucleotides and engage effectors, but they differ in their C-terminal membrane targeting sequences, designated hypervariable regions (HVRs), that mediate subcellular trafficking and membrane association (Tsai et al., 2015) (Figure 1). Membrane targeting is accomplished by posttranslational modification (PTM) of the HVR converting nascent RAS from a globular, hydrophilic protein to a peripheral membrane protein with a hydrophobic C-terminus that affords affinity for phospholipid bilayers (Wright and Philips, 2006) (Figure 1). Notably, both gene products are oncogenic when the *KRAS* locus sustains a mutation in exons 2 or 3 resulting in constitutively active KRAS proteins, each capable of transforming cells (Voice et al., 1999), suggesting that studying the characteristics of both isoforms will provide a better understanding of the biology of KRAS-driven cancers (Figure 1). As a result of constitutive activation, RAS-mediated signaling cascades drive tumor onset, maintenance, and progression by deregulating key cellular processes (Hobbs et al., 2016), altering the tumor microenvironment (Pylayeva-Gupta et al., 2011), and rewiring cellular metabolism to an enhanced glycolysis (Mukhopadhyay et al., 2021). Whereas KRAS4B is the most studied of all oncoproteins, KRAS4A is understudied and, until recently, considered relatively unimportant. Emerging work has confirmed expression of KRAS4A in cancer and found non-overlapping functions of the splice variants. Thus, understanding the biochemical and cell biological properties of *KRAS* splice variants is required to fully explore vulnerabilities that can be exploited for drug discovery.

## Membrane association and trafficking of KRAS proteins

The *KRAS* transcript undergoes alternative splicing giving rise to two 21 kDa isoforms: KRAS4A and KRAS4B. Both proteins share nearly identical G domains consisting of the first 165 aa. The 5’ portion of exon 4 encodes the final segment of the G domain preceding the 23 aa HVR. This gives rise to 3 G domain differences at residues 151, 153 and 165 (Laude and Prior, 2008; Raso, 2020) (Figure 1). The G domain is the catalytic and switching portion of the protein that binds GDP/GTP and associates with effectors, exchange factors, and GTPase-activating proteins (GAPs) (Vigil et al., 2010). However, KRAS splice variants differ substantially in their HVRs which contain the targeting information for the membrane localization that is necessary for RAS function (Willumsen et al., 1984; Laude and Prior, 2008; Tsai et al., 2015). The HVR includes a C-terminal CaaX sequence, which is modified posttranslationally in three steps: farnesylation, aaX proteolysis, and carboxyl methylation of the resulting C-terminal prenylcysteine (Wright and Philips, 2006) (Figure 1). All RAS proteins other than KRAS4B also possess one or two cysteines immediately upstream of the CaaX sequence that are modified with palmitate. KRAS4B lacks a palmitoylation site and instead incorporates a polylysine sequence that can form an electrostatic interaction with the negatively charged inner leaflet of the plasma membrane (Laude and Prior, 2008; Jang et al., 2015) (Figure 1). The PTMs allow RAS to associate with membranes by remodeling the carboxyl terminus, a feature that is required for signaling and therefore, RAS function (Ahearn et al., 2018). Thus, the two *KRAS* splice variants have distinct mechanisms of subcellular trafficking. KRAS4A is unique in possessing a dual membrane-targeting motif since it is not only palmitoylated but also contains two short polybasic regions (PBR) (Tsai et al., 2015; Ahearn et al., 2018) (Figure 1). Palmitoylation occurs on cysteine 180 in the C-terminal membrane-targeting region independently mediating an efficient plasma membrane association (Tsai et al., 2015). Both palmitoylation and the PBRs are required for maximal signaling efficiency and mutation of either PBR combined with loss of palmitoylation cause a significant reduction in ERK phosphorylation (Tsai et al., 2015), which has also been demonstrated to abolish the ability of oncogenic KRAS4A to induce leukemia in mice (Zhao et al., 2015). The distinct membrane anchors of KRAS4A and KRAS4B predict differential dynamics with regard to membrane association. Indeed, using single-molecule-tracking to assess RAS mobility, it was observed that KRAS4B exhibits a distinct behavior when compared to the other RAS proteins (Goswami et al., 2020). Mechanistically, the authors proposed that the positive charges on the HVR of KRAS4B recruit and cluster negatively charged lipids around RAS molecules, and subsequent G domain interactions with both effectors and lipids lead to an ordered, stepwise assembly process where KRAS4B molecules are increasingly confined to smaller nanodomains in the formation of a signaling complex (Goswami et al., 2020).

Another unique feature of KRAS4B is the presence of a phosphorylation site on serine 181 that behaves as a farnesyl electrostatic switch, weakening association with the plasma membrane and promoting translocation to other endomembrane compartments (Bivona et al., 2006; Barcelo et al., 2014). Binding of calmodulin to KRAS4B has been shown to block phosphorylation of serine 181. Interestingly, calmodulin binding suppresses noncanonical Wnt signaling induced by oncogenic KRAS4B (Wang et al., 2015; Sperlich et al., 2016). Disruption of this interaction has been proposed to reduce the tumorigenic properties of mutant KRAS4B in pancreatic cancer (Wang et al., 2015).

PDE6δ is a prenyl binding protein that can act as a cytosolic chaperone (Chandra et al., 2011). PDE6δ has been co-crystalized with prenylated KRAS4B confirming the interaction (Dharmaiah et al., 2016). Whereas overexpression of PDE6δ can extract KRAS4B from membranes (Dharmaiah et al., 2016) demonstrating interaction in living cells, it does not affect the localization KRAS4A, highlighting a significant difference in the subcellular trafficking of the splice variants (Tsai et al., 2015).

The Lin group demonstrated that KRAS4A is subject to lysine fatty acylation at its C-terminal HVR (Jing et al., 2017). Sirtuin 2 (SIRT2), one of the mammalian nicotinamide adenine dinucleotide (NAD)-dependent lysine deacylases, catalyzes the removal of fatty acylation from KRAS4A leading to an increased endomembrane localization, interaction with A-Raf, and enhanced KRAS4A transforming activity, suggesting that SIRT2 may be therapeutic target for treating cancers associated with KRAS4A expression (Jing et al., 2017). In this regard, the group developed a SIRT2 inhibitor, JH-T4, a small molecule that successfully modulates the lysine fatty acylation levels of KRAS4A and was cytotoxic to several human cancer cells. However, the cytotoxic effect was not selective to cancer cells. According to the authors, this phenomenon might be due to ability of JH-T4 to inhibit other sirtuins or due to inhibition of the defatty-acylation activity of SIRT2 (Spiegelman et al., 2019).

The differential modification and trafficking of the two *KRAS* splice variants has prompted studies designed to determine if the two proteins have distinct interactomes. The Lin group used stable isotope labelling with amino acids in cell culture (SILAC) and affinity-purification mass spectrometry (AP-MS) to examine the nucleotide-dependent interactomes of KRAS4A and KRAS4B (Zhang et al., 2018), and later applied the same technology to nucleotide-dependent interactomes of the four RAS isoforms, KRAS4A, KRAS4B, HRAS, and NRAS (Miller et al., 2022). Although the majority of the proteins identified in these studies need to be further validated, the investigators were able to draw some interesting conclusions. For example, they found that v-ATPase a2 interacts only with KRAS4B, an interaction that may have physiological relevance since the V-ATPase has been shown to regulate KRAS-induced micropinocytosis (Ramirez et al., 2019). Moreover, the interaction occurs on the cytosolic face of lysosomes, consistent with the observation that KRAS4B but not KRAS4A is localized on lysosomes (Zhang et al., 2018). These results suggested that the different intracellular localizations of KRAS4A and KRAS4B may explain differential protein-protein interactions (Zhang et al., 2018). This concept was validated by the discovery that KRAS4A but not KRAS4B associates with hexokinase 1 (HK1) on the outer mitochondrial membrane (OMM) and that the basis for the differential association was due entirely to the ability of depalmitoyated KRAS4A to traffic to the OMM (Amendola et al., 2019). Importantly, KRAS4A was shown to stimulate the activity of HK1, thereby directly regulating cancer cells metabolism (see below) (Amendola et al., 2019).

## KRAS splice variants and glycolysis

**FIGURE 2:**
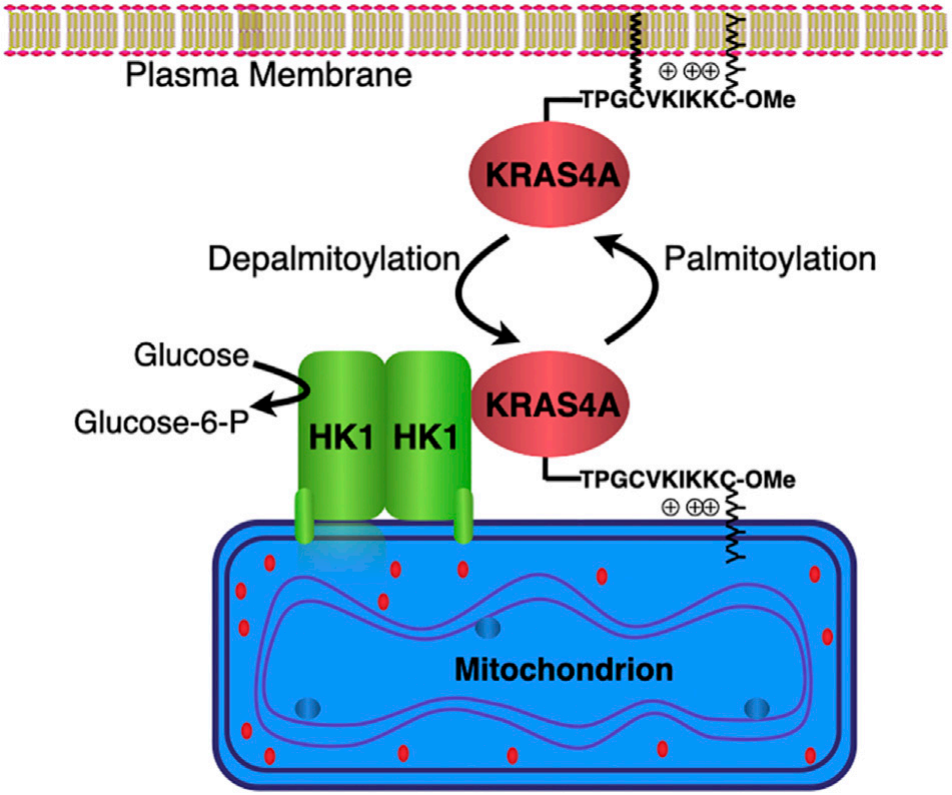
KRAS4A regulates hexokinase 1 catalytic activity. Hexokinase 1 (HK1) is resident on the outer mitochondrial membrane (OMM) owing to an N-terminal targeting sequence. KRAS4A undergoes a cycle of palmitoylation/depalmitoylation on cysteine 180 that drives association with the plasma membrane or OMM, respectively. KRAS4A interacts with HK1 on the OMM in a GTP-dependent fashion and stimulates HK1 activity by limiting the allosteric feedback inhibition physiologically mediated by glucose-6-phosphate.

RAS-mediated signaling drives tumor onset, maintenance and progression not only by deregulating tumor-autonomous cellular growth control and altering the tumor microenvironment (Pylayeva-Gupta et al., 2011; Simanshu et al., 2017; Hamarsheh et al., 2020), but also by rewiring cellular metabolism to enhance glycolysis (Ying et al., 2012; Mukhopadhyay et al., 2021). The reprogramming of cellular metabolism to provide the energetic and biomass demands of uncontrolled cell proliferation is one of the hallmarks of cancer (Hanahan and Weinberg, 2011). In this regard, one of the most common metabolic characteristics of cancer cells is an altered glucose metabolism known as the Warburg effect, in which cancer cells engage in enhanced glycolysis even in the presence of abundant oxygen (Warburg, 1956). As a result of the breakdown of glucose through glycolysis, cancer cells produce ATP and metabolic intermediates required for cell growth and proliferation (Hsu and Sabatini, 2008). Oncogenic KRAS increases glycolysis and shunts glycolytic intermediated to specific anabolic pathways (Kimmelman, 2015). In particular, KRAS has been implicated in the regulation of the expression of rate-limiting metabolic enzymes and to the shunt of glucose-derived metabolites to the nonoxidative arm of the pentose phosphate pathway to promote ribose biosynthesis (Kimmelman, 2015). The effects on glycolysis reported by Kimmelman and others were understood as an indirect effect mediated by transcriptional upregulation of glycolytic enzymes (Yun et al., 2009; Ying et al., 2012; Bryant et al., 2014). More recently, a direct effect of KRAS on glycolysis has been established and shown to be specific for KRAS4A (Amendola et al., 2019) (Figure 2).

Using affinity purification and mass spectroscopy, Amendola et al. demonstrated a direct interaction between KRAS4A and HK1, establishing the first such interaction between a RAS protein and a metabolic enzyme (Amendola et al., 2019). The interaction was GTP-dependent and enhanced when palmitoylation of KRAS4A was blocked. Depalmitoylated KRAS4A colocalized with HK1 on the OMM (Figure 2). When HRAS was artificially targeted to the OMM it too interacted with HK1, demonstrating that the isoform-specificity was driven entirely by subcellular localization. Importantly, the interaction increased the enzymatic activity of HK1, thus establishing the enzyme as a bona fide effector of KRAS4A. Mechanistically, GTP-bound KRAS4A stimulated HK1 catalytic activity by limiting the allosteric feedback inhibition physiologically mediated by glucose-6-phosphate (Figure 2). In fact, cell-based studies revealed a diminished glycolytic flux in *KRAS* mutant tumor cells in which *KRAS4A* was silenced with CRISPR/Cas9 (Amendola et al., 2019). Furthermore, xenograft tumors produced with these cells showed dramatically diminished glucose uptake by [^18^F]-DG PET scanning relative to parental cells (Amendola et al., 2019).

*RAS*-mutant tumors are among the most aggressive and refractory to treatment (Hymowitz and Malek, 2018; Punekar et al., 2022). Although some success in developing direct inhibitors of KRAS has been made recently (Basso et al., 2006; Engelman et al., 2008; Cox et al., 2015; McCormick, 2015; Punekar et al., 2022), disappointing clinical trials of these agents (Hu et al., 2000; Basso et al., 2006; Berndt et al., 2011; Riely et al., 2011; Gelderblom et al., 2020) (NCT01468688, NCT01297491, NCT03101839, NCT04111458) demonstrate that a multi-pronged approach will be required to have a significant therapeutic impact. The recent establishment of differential effects on metabolism of the two KRAS splice variants suggest the possibility of exploiting therapeutically unique metabolic vulnerabilities in cancers with relatively high expression of the KRAS4A.

## Relative expression of KRAS splice variants

KRAS has been intensively studied for more than 4 decades but the vast majority of work has focused on KRAS4B. Because of lower levels of expression, KRAS4A was considered the “minor” splice variant and largely ignored. Ironically, KRAS4A proved to be the transforming gene carried by the Kirsten murine sarcoma virus from which the name “K”RAS derives (Shimizu et al., 1983). It has been shown conclusively that the expression levels of KRAS4A and KRAS4B vary profoundly across different tissues and developmental stages (Tsai et al., 2015). As early as 1983 a 20-fold lower level of *KRAS4A* compared to *KRAS4B* transcript was reported from various cancer cell lines using RNA hybridization (Capon et al., 1983). Using the polymerase colony method to analyze the splice variants abundance, Butz, Roberta and Edwards found a 9.6-fold difference of (*KRAS4B*:*KRAS4A*) in SW480 cells and a 47-fold difference in PANC-1 cells compared to 1.4-fold difference in normal human colon, suggesting that expression of *KRAS4A* is lost in cancer (Butz et al., 2004). Consistent with these data, using isotopic peptide standards Prior and colleagues measured the absolute abundance of RAS proteins in a panel of isogenic SW48 colorectal cancer cells and found no KRAS4A-specific peptides (Mageean et al., 2015). However, a study of colorectal cancer cases in Saudi Arabian patients not only detected KRAS4A transcript but reported a correlation between KRAS4A overexpression and better overall survival and cleaved caspase-3 expression, consistent with other reports describing a pro-apoptotic function for KRAS4A (Abubaker et al., 2009). Another study showed that KRAS4A was expressed in both human renal cell carcinomas and human renal cell carcinoma cells lines, with its upregulation associated with sensitivity to aldosterone (King et al., 2014). Using northern blot hybridization, the expression of KRAS4A and KRAS4B transcripts was assessed in 8 different mouse organs including heart, brain, spleen, lung, liver, skeletal muscle, kidney, and testis. KRAS4B mRNA was found in all 8 organs representing approximately 90%–99% of total KRAS mRNA meanwhile KRAS4A mRNA was detected only in lung, liver, and kidney (Wang et al., 2001). In another study, KRAS4A expression was explored in human tissues and colorectal tumors (Plowman et al., 2006b). Using RT-PCR with concurrent metabolic labeling of PCR product with [^33^P]dATP and quantification from autoradiography after PAGE analysis, KRAS4A was found to be expressed primarily in the gastrointestinal tract, though it was also detected in kidney, lung, bone marrow, and other tissues (Plowman et al., 2006b). More recently Tsai et al. employed PCR primers specific for splice junctions and cDNA templates that allowed creating standard curves and quantifying absolute levels of mRNA to more accurately measure relative levels of transcript (Tsai et al., 2015). KRAS4A mRNA was present in all 30 human cell lines examined. Although KRAS4B message was present at higher levels in most of these cells, in 40% of them, the KRAS4A transcript exceeded 20% of total KRAS message, and in several, the mRNA levels for the two splice variants were similar. Using a KRAS4A-specific antibody these results were validated at the protein level. Importantly, the analysis of fresh colorectal tumors showed that the expression levels of the two transcripts were equal over a panel of 17 samples (Tsai et al., 2015). A subsequent study using qRT-PCR measured the gene expression profiles of each of the RAS isoforms in a panel of mouse tissues derived from a full developmental time course spanning embryogenesis through to adulthood showing a relative contribution of KRAS4B > > NRAS ≥ KRAS4A > HRAS to total RAS expression with KRAS4B typically representing 60–99% of all *RAS* transcripts (Newlaczyl et al., 2017). Interestingly, the authors showed that KRAS4A is the most dynamically regulated RAS isoform with significant up-regulation of expression observed pre-term in stomach, intestine, kidney, and heart (Newlaczyl et al., 2017). More recently, using qRT-PCR Aran and colleagues found that the *KRAS4B* transcript was two-fold higher than that of *KRAS4A* in 55 samples of advanced non-small cell lung cancer (NSCLC) but relative to normal lung tissue *KRAS4A* message was elevated in 42 of 55 patients (Aran et al., 2018). These authors also reported enhanced KRAS4A protein using immunohistochemistry (IHC), however no controls were shown and no commercial antibody has been validated for detection of endogenous RAS by IHC (Waters et al., 2017). Another study of lung adenocarcinoma (LUAD) patients analyzed RNA expression and somatic mutation data from The Cancer Genome Atlas (n = 516) to assess the overall survival (OS) and disease-free survival (DFS) based on the abundance of *KRAS* transcript variants (Yang and Kim, 2018). In this work, the authors found that the expression of the *KRAS4A* transcript was positively correlated with the presence of *KRAS* mutations and associated with poor OS and DFS in LUAD patients (Yang and Kim, 2018). Using splicing-sensitive microarrays and RNA sequencing, the abundance of both *KRAS* splice variants was investigated in samples from patients with microsatellite stable (MSS) colorectal cancer (CRC) (Eilertsen et al., 2019). Eilertsen et al. found that aberrant splicing resulting in low expression of the *KRAS4A* transcript variant, in relation to the *KRAS4B* transcript, was associated with increased KRAS signaling and poor patient prognosis specifically in KRAS wild-type MSS CRC suggesting that *KRAS* splicing is of prognostic relevance in KRAS wild-type CRC (Eilertsen et al., 2019).

The relative abundance of the KRAS splice variants is undoubtedly controlled by the efficiency of alternative splicing. The regulation of the alternative splicing of the *KRAS* transcript remains relatively unexplored. Balmain and colleagues reported that *KRAS4A* splicing is controlled by the DCAF15/RBM39 pathway, and deletion of KRAS4A or pharmacological inhibition of RBM39 using the splicing inhibitor Indisulam leads to inhibition of cancer stem cells (Chen et al., 2021). Overall, these data suggest that levels of KRAS4A isoform in human tumors can be a biomarker of sensitivity to some existing cancer therapeutics.

## KRAS splice variants in development and cancer biology

Although their relative expression in normal tissues and cancer is not completely understood, efforts have been made to better understand the role of both splice variants in cancer biology. In 1999 it was reported that mutationally activated KRAS4A was able to activate RAF1 and signal through the MAPK pathway more efficiently than oncogenic *HRAS* or *NRAS* (Voice et al., 1999). Furthermore, in this study oncogenic KRAS4A induced the formation of transformed foci and enable anchorage-independent growth significantly better than oncogenic KRAS4B expressed at the same level (Voice et al., 1999). These pioneering observations suggested an important role for KRAS4A in carcinogenesis. More nuanced results have been obtained from the study of genetically engineered mice (GEM). Interestingly, whereas the *Kras* locus is essential for mouse development, *Nras* and *Hras* loci are dispensable (Johnson et al., 1997; Koera et al., 1997; Esteban et al., 2001). However, mice homozygous for the *Hras* cDNA knocked into the *Kras* locus are viable, despite being afflicted with dilated cardiomyopathy and arterial hypertension later in life (Potenza et al., 2005). This suggests that the *Kras* locus might be essential partially because of gene expression patterns rather than differential function of the gene products. Like *Hras* and *Nras*, KRAS4A is dispensable for normal mouse development, at least in the presence of functional KRAS4B (Plowman et al., 2003). Using embryonic stem cells derived from the *Kras4a* knock-out animal, these authors concluded that whereas KRAS4A promotes apoptosis, KRAS4B inhibits it, and that both KRAS4A and KRAS4B promote differentiation (Plowman et al., 2003). These findings raise the possibility that alteration of the KRAS4A/KRAS4B isoform ratio modulates tumorigenesis by differentially affecting stem cell survival and/or differentiation, in agreement with previous observations related to both isoforms (Voice et al., 1999). However, *Kras4a* deficiency did not affect life expectancy or spontaneous overall tumor incidence in aging mice (Plowman et al., 2006a). Similarly, when colonic adenomas were induced in mice lacking *Kras4a*, an increase in number and size of colonic adenomas was observed that revealed increased markers of proliferation and decreased markers of apoptosis (Luo et al., 2010). However, a similar study using a GEM to induce colon carcinomas showed no such effect of *Kras4a* deficiency (Patek et al., 2008). Another study using *Kras* knockout mouse embryonic fibroblasts showed that the transcription of matrix metalloproteinase 2 (MMP-2) was lost in these cells and partially restored by transient expression of KRAS4B but not KRAS4A, suggesting a steady-state regulation of MMP-2 expression by KRAS4B (Liao et al., 2003). However, the effect of selective elimination of one or the other splice variant was not studied. Recently, a *Kras4B*-null GEM has been generated selectively disrupting the expression of the KRAS4B isoform by inserting a premature termination codon into its coding sequence giving rise to a highly unstable truncated protein without affecting either transcription or translation of the KRAS4A isoform (Salmon et al., 2021). In this work, the authors found no postnatal development in the absence of KRAS4B. Hence, the expression of the endogenous KRAS4B isoform, in contrast to KRAS4A, is strictly required for mouse development. Interestingly, expression of endogenous KRAS4A^G12V^ in the absence of KRAS4B was sufficient to induce lung adenocarcinomas that undergo proximal metastasis (Salmon et al., 2021). Intriguingly, using a model of liver transgenesis, mutant KRAS4A induced higher ERK1/2 phosphorylation resulting in increased expression of p16 and consequently fewer tumors suggesting that lower levels of mutant KRAS4A, as compared with those of KRAS4B, are sufficient to induce tumorigenesis *in vivo* (Chung et al., 2016; Salmon et al., 2021).

Recently, the Balmain group developed a *Kras4B* ^−/−^ mouse model by inserting a *Kras4a* cDNA into the *Kras* locus (Potenza et al., 2005; Chen et al., 2021) with the aim of comparing the roles of KRAS4A and KRAS4B in lung tumorigenesis. Strikingly, these authors found that coordinated regulation of both isoforms is essential for development of *Kras* mutant tumors (Chen et al., 2021). Previous reports had shown that KRAS4A is expressed during differentiation of pluripotent embryonic stem cells and in a subset of cells in adult tissues (Pells et al., 1997), raising the possibility that KRAS4A has specific functions in a small population of cells with stem cell properties (To et al., 2008). In line with these observations, Balmain and colleagues showed that human KRAS4A, but not KRAS4B, is enriched in stem cell-like side population cells derived from human cancer cell lines (Chen et al., 2021). Loss of KRAS4A, but not KRAS4B, causes a decrease in the proportion of cells with side population characteristics. In addition, hypoxic conditions which are known to lead to reactivation of stem cells, cause an upregulation of KRAS4A, but not KRAS4B suggesting that the splicing of *KRAS* to generate the 4A and 4B isoforms may be a critical event in controlling stress responses and the proliferative or metabolic requirements in stem and progenitor cells (Chen et al., 2021). On the other hand, expression of KRAS4B but not KRAS4A was induced by endoplasmic reticulum (ER) stress (Chen et al., 2021), which has been previously identified as a therapeutic target in *KRAS* mutant cancers (De Raedt et al., 2011). In summary, although evidence for differential roles of the *KRAS* splice variants in development and oncogenesis has been reported, future studies will be needed to better understand the roles of KRAS4A and KRAS4B.

## Final remarks

Whereas KRAS4B is the most studied oncoprotein, KRAS4A is understudied and until recently considered relatively unimportant. Despite evolutionary conservation over hundreds of millions of years of the alternate splicing of the *KRAS* transcript and some splice-variant specific interactions reported in mass spec screens, no biochemical/signaling difference had been established until the recent discovery that KRAS4A but not KRAS4B interacts with and regulates HK1. This work showed that cancer cells metabolism can be modulated as a consequence of unique subcellular trafficking of KRAS4A (Amendola et al., 2019) (Figure 2). This highly significant finding establishes for the first time a direct effect of RAS on metabolism, represents the first demonstration of a biochemical difference between *KRAS* splice variants, and suggests a unique vulnerability for *KRAS* driven cancers that express oncogenic KRAS4A. Given that *KRAS* is mutated in human cancer more frequently than any other oncogene, the two products of the *KRAS* locus and the pathways they regulate are among the most attractive targets for anti-cancer drug discovery. Elucidation of the differential consequences of the expression of the splice variants will inform the development of therapeutics designed to block *KRAS* driven cancer.

## References

[B1] Abubaker J. Bavi P. Al-Haqawi W. Sultana M. Al-Harbi S. Al-Sanea N. (2009). Prognostic significance of alterations in KRAS isoforms KRAS-4A/4B and KRAS mutations in colorectal carcinoma. J. Pathol. 219 (4), 435–445. 10.1002/path.2625 19824059

[B2] Ahearn I. M. Haigis K. Bar-Sagi D. Philips M. R. (2011). Regulating the regulator: Post-translational modification of RAS. Nat. Rev. Mol. Cell Biol. 13 (1), 39–51. 10.1038/nrm3255 22189424 PMC3879958

[B3] Ahearn I. Zhou M. Philips M. R. (2018). Posttranslational modifications of RAS proteins. Cold Spring Harb. Perspect. Med. 8 (11), a031484. 10.1101/cshperspect.a031484 29311131 PMC6035883

[B4] Amendola C. R. Mahaffey J. P. Parker S. J. Ahearn I. M. Chen W. C. Zhou M. (2019). KRAS4A directly regulates hexokinase 1. Nature 576 (7787), 482–486. 10.1038/s41586-019-1832-9 31827279 PMC6923592

[B5] Aran V. Masson Domingues P. Carvalho de Macedo F. Moreira de Sousa C. A. Caldas Montella T. de Souza Accioly M. T. (2018). A cross-sectional study examining the expression of splice variants K-RAS4A and K-RAS4B in advanced non-small-cell lung cancer patients. Lung Cancer 116, 7–14. 10.1016/j.lungcan.2017.12.005 29413054

[B6] Barcelo C. Paco N. Morell M. Alvarez-Moya B. Bota-Rabassedas N. Jaumot M. (2014). Phosphorylation at Ser-181 of oncogenic KRAS is required for tumor growth. Cancer Res. 74 (4), 1190–1199. 10.1158/0008-5472.CAN-13-1750 24371225

[B7] Basso A. D. Kirschmeier P. Bishop W. R. (2006). Lipid posttranslational modifications. Farnesyl transferase inhibitors. J. Lipid Res. 47 (1), 15–31. 10.1194/jlr.R500012-JLR200 16278491

[B8] Berndt N. Hamilton A. D. Sebti S. M. (2011). Targeting protein prenylation for cancer therapy. Nat. Rev. Cancer 11 (11), 775–791. 10.1038/nrc3151 22020205 PMC4037130

[B9] Bivona T. G. Quatela S. E. Bodemann B. O. Ahearn I. M. Soskis M. J. Mor A. (2006). PKC regulates a farnesyl-electrostatic switch on K-Ras that promotes its association with Bcl-XL on mitochondria and induces apoptosis. Mol. Cell 21 (4), 481–493. 10.1016/j.molcel.2006.01.012 16483930

[B10] Bryant K. L. Mancias J. D. Kimmelman A. C. Der C. J. (2014). Kras: Feeding pancreatic cancer proliferation. Trends biochem. Sci. 39 (2), 91–100. 10.1016/j.tibs.2013.12.004 24388967 PMC3955735

[B11] Butz J. A. Roberts K. G. Edwards J. S. (2004). Detecting changes in the relative expression of KRAS2 splice variants using polymerase colonies. Biotechnol. Prog. 20 (6), 1836–1839. 10.1021/bp0343054 15575719

[B12] Capon D. J. Seeburg P. H. McGrath J. P. Hayflick J. S. Edman U. Levinson A. D. (1983). Activation of Ki-ras2 gene in human colon and lung carcinomas by two different point mutations. Nature 304 (5926), 507–513. 10.1038/304507a0 6308467

[B13] Chandra A. Grecco H. E. Pisupati V. Perera D. Cassidy L. Skoulidis F. (2011). The GDI-like solubilizing factor PDEδ sustains the spatial organization and signalling of Ras family proteins. Nat. Cell Biol. 14 (2), 148–158. 10.1038/ncb2394 22179043

[B14] Chen W. C. To M. D. Westcott P. M. K. Delrosario R. Kim I. J. Philips M. (2021). Targeting KRAS4A splicing through the RBM39/DCAF15 pathway inhibits cancer stem cells. Nat. Commun. 12 (1), 4288. 10.1038/s41467-021-24498-7 34257283 PMC8277813

[B15] Chiu V. K. Bivona T. Hach A. Sajous J. B. Silletti J. Wiener H. (2002). Ras signalling on the endoplasmic reticulum and the Golgi. Nat. Cell Biol. 4 (5), 343–350. 10.1038/ncb783 11988737

[B16] Chung S. I. Moon H. Ju H. L. Kim D. Y. Cho K. J. Ribback S. (2016). Comparison of liver oncogenic potential among human RAS isoforms. Oncotarget 7 (6), 7354–7366. 10.18632/oncotarget.6931 26799184 PMC4872791

[B17] Cox A. D. Der C. J. Philips M. R. (2015). Targeting RAS membrane association: Back to the future for anti-RAS drug discovery? Clin. Cancer Res. 21 (8), 1819–1827. 10.1158/1078-0432.CCR-14-3214 25878363 PMC4400837

[B18] Cox A. D. Der C. J. (2010). Ras history: The saga continues. Small GTPases 1 (1), 2–27. 10.4161/sgtp.1.1.12178 21686117 PMC3109476

[B19] De Raedt T. Walton Z. Yecies J. L. Li D. Chen Y. Malone C. F. (2011). Exploiting cancer cell vulnerabilities to develop a combination therapy for ras-driven tumors. Cancer Cell 20 (3), 400–413. 10.1016/j.ccr.2011.08.014 21907929 PMC3233475

[B20] Dharmaiah S. Bindu L. Tran T. H. Gillette W. K. Frank P. H. Ghirlando R. (2016). Structural basis of recognition of farnesylated and methylated KRAS4b by PDEδ. Proc. Natl. Acad. Sci. U. S. A. 113 (44), E6766-E6775–E6775. 10.1073/pnas.1615316113 27791178 PMC5098621

[B21] Eilertsen I. A. Sveen A. Stromme J. M. Skotheim R. I. Nesbakken A. Lothe R. A. (2019). Alternative splicing expands the prognostic impact of KRAS in microsatellite stable primary colorectal cancer. Int. J. Cancer 144 (4), 841–847. 10.1002/ijc.31809 30121958 PMC6587976

[B22] Engelman J. A. Chen L. Tan X. Crosby K. Guimaraes A. R. Upadhyay R. (2008). Effective use of PI3K and MEK inhibitors to treat mutant Kras G12D and PIK3CA H1047R murine lung cancers. Nat. Med. 14 (12), 1351–1356. 10.1038/nm.1890 19029981 PMC2683415

[B23] Esteban L. M. Vicario-Abejon C. Fernandez-Salguero P. Fernandez-Medarde A. Swaminathan N. Yienger K. (2001). Targeted genomic disruption of H-ras and N-ras, individually or in combination, reveals the dispensability of both loci for mouse growth and development. Mol. Cell. Biol. 21 (5), 1444–1452. 10.1128/MCB.21.5.1444-1452.2001 11238881 PMC86690

[B24] Gelderblom H. Jones R. L. George S. Valverde Morales C. Benson C. Jean-Yves B. (2020). Imatinib in combination with phosphoinositol kinase inhibitor buparlisib in patients with gastrointestinal stromal tumour who failed prior therapy with imatinib and sunitinib: A phase 1b, multicentre study. Br. J. Cancer 122 (8), 1158–1165. 10.1038/s41416-020-0769-y 32147671 PMC7156686

[B25] Goswami D. ChenYang Y. Gudla P. R. Columbus J. Worthy K. Rigby M. (2020). Membrane interactions of the globular domain and the hypervariable region of KRAS4b define its unique diffusion behavior. Elife 9, e47654. 10.7554/eLife.47654 31958057 PMC7060043

[B26] Hamarsheh S. Gross O. Brummer T. Zeiser R. (2020). Immune modulatory effects of oncogenic KRAS in cancer. Nat. Commun. 11 (1), 5439. 10.1038/s41467-020-19288-6 33116132 PMC7595113

[B27] Hanahan D. Weinberg R. A. (2011). Hallmarks of cancer: The next generation. Cell 144 (5), 646–674. 10.1016/j.cell.2011.02.013 21376230

[B28] Hobbs G. A. Der C. J. Rossman K. L. (2016). RAS isoforms and mutations in cancer at a glance. J. Cell Sci. 129 (7), 1287–1292. 10.1242/jcs.182873 26985062 PMC4869631

[B29] Hsu P. P. Sabatini D. M. (2008). Cancer cell metabolism: Warburg and beyond. Cell 134 (5), 703–707. 10.1016/j.cell.2008.08.021 18775299

[B30] Hu L. Zaloudek C. Mills G. B. Gray J. Jaffe R. B. (2000). *In vivo* and *in vitro* ovarian carcinoma growth inhibition by a phosphatidylinositol 3-kinase inhibitor (LY294002). Clin. Cancer Res. 6 (3), 880–886. 10741711

[B31] Hymowitz S. G. Malek S. (2018). Targeting the MAPK pathway in RAS mutant cancers. Cold Spring Harb. Perspect. Med. 8 (11), a031492. 10.1101/cshperspect.a031492 29440321 PMC6211377

[B32] Jang H. Abraham S. J. Chavan T. S. Hitchinson B. Khavrutskii L. Tarasova N. I. (2015). Mechanisms of membrane binding of small GTPase K-Ras4B farnesylated hypervariable region. J. Biol. Chem. 290 (15), 9465–9477. 10.1074/jbc.M114.620724 25713064 PMC4392252

[B33] Jing H. Zhang X. Wisner S. A. Chen X. Spiegelman N. A. Linder M. E. (2017). SIRT2 and lysine fatty acylation regulate the transforming activity of K-Ras4a. Elife 6, e32436. 10.7554/eLife.32436 29239724 PMC5745086

[B34] Johnson L. Greenbaum D. Cichowski K. Mercer K. Murphy E. Schmitt E. (1997). K-ras is an essential gene in the mouse with partial functional overlap with N-ras. Genes Dev. 11 (19), 2468–2481. 10.1101/gad.11.19.2468 9334313 PMC316567

[B35] Kiel C. Matallanas D. Kolch W. (2021). The ins and outs of RAS effector complexes. Biomolecules 11 (2), 236. 10.3390/biom11020236 33562401 PMC7915224

[B36] Kimmelman A. C. (2015). Metabolic dependencies in RAS-driven cancers. Clin. Cancer Res. 21 (8), 1828–1834. 10.1158/1078-0432.CCR-14-2425 25878364 PMC4400826

[B37] King S. Bray S. Galbraith S. Christie L. Fleming S. (2014). Evidence for aldosterone-dependent growth of renal cell carcinoma. Int. J. Exp. Pathol. 95 (4), 244–250. 10.1111/iep.12074 24802662 PMC4170966

[B38] Koera K. Nakamura K. Nakao K. Miyoshi J. Toyoshima K. Hatta T. (1997). K-ras is essential for the development of the mouse embryo. Oncogene 15 (10), 1151–1159. 10.1038/sj.onc.1201284 9294608

[B39] Laude A. J. Prior I. A. (2008). Palmitoylation and localisation of RAS isoforms are modulated by the hypervariable linker domain. J. Cell Sci. 121 (Pt 4), 421–427. 10.1242/jcs.020107 18211960

[B40] Liao J. Wolfman J. C. Wolfman A. (2003). K-ras regulates the steady-state expression of matrix metalloproteinase 2 in fibroblasts. J. Biol. Chem. 278 (34), 31871–31878. 10.1074/jbc.M301931200 12805379

[B41] Luo F. Ye H. Hamoudi R. Dong G. Zhang W. Patek C. E. (2010). K-ras exon 4A has a tumour suppressor effect on carcinogen-induced murine colonic adenoma formation. J. Pathol. 220 (5), 542–550. 10.1002/path.2672 20087880

[B42] Mageean C. J. Griffiths J. R. Smith D. L. Clague M. J. Prior I. A. (2015). Absolute quantification of endogenous ras isoform abundance. PLoS One 10 (11), e0142674. 10.1371/journal.pone.0142674 26560143 PMC4641634

[B43] McCormick F. (2015). KRAS as a therapeutic target. Clin. Cancer Res. 21 (8), 1797–1801. 10.1158/1078-0432.CCR-14-2662 25878360 PMC4407814

[B44] Miller S. P. Maio G. Zhang X. Badillo Soto F. S. Zhu J. Ramirez S. Z. (202210026). A proteomic approach identifies isoform-specific and nucleotide-dependent RAS interactions. Mol. Cell. Proteomics. 21, 100268. 10.1016/j.mcpro.2022.100268 PMC9396065 35839996

[B45] Mor A. Philips M. R. (2006). Compartmentalized ras/MAPK signaling. Annu. Rev. Immunol. 24, 771–800. 10.1146/annurev.immunol.24.021605.090723 16551266

[B46] Mukhopadhyay S. Vander Heiden M. G. McCormick F. (2021). The metabolic landscape of RAS-driven cancers from biology to therapy. Nat. Cancer 2, 271–283. 10.1038/s43018-021-00184-x 33870211 PMC8045781

[B47] Newlaczyl A. U. Coulson J. M. Prior I. A. (2017). Quantification of spatiotemporal patterns of Ras isoform expression during development. Sci. Rep. 7, 41297. 10.1038/srep41297 28117393 PMC5259795

[B48] Patek C. E. Arends M. J. Wallace W. A. Luo F. Hagan S. Brownstein D. G. (2008). Mutationally activated K-ras 4A and 4B both mediate lung carcinogenesis. Exp. Cell Res. 314 (5), 1105–1114. 10.1016/j.yexcr.2007.11.004 18062963

[B49] Pells S. Divjak M. Romanowski P. Impey H. Hawkins N. J. Clarke A. R. (1997). Developmentally-regulated expression of murine K-ras isoforms. Oncogene 15 (15), 1781–1786. 10.1038/sj.onc.1201354 9362444

[B50] Plowman S. J. Arends M. J. Brownstein D. G. Luo F. Devenney P. S. Rose L. (2006a). The K-Ras 4A isoform promotes apoptosis but does not affect either lifespan or spontaneous tumor incidence in aging mice. Exp. Cell Res. 312 (1), 16–26. 10.1016/j.yexcr.2005.10.004 16271715

[B51] Plowman S. J. Berry R. L. Bader S. A. Luo F. Arends M. J. Harrison D. J. (2006b). K-ras 4A and 4B are co-expressed widely in human tissues, and their ratio is altered in sporadic colorectal cancer. J. Exp. Clin. Cancer Res. 25 (2), 259–267. 16918139

[B52] Plowman S. J. Williamson D. J. O'Sullivan M. J. Doig J. Ritchie A. M. Harrison D. J. (2003). While K-ras is essential for mouse development, expression of the K-ras 4A splice variant is dispensable. Mol. Cell. Biol. 23 (24), 9245–9250. 10.1128/MCB.23.24.9245-9250.2003 14645534 PMC309700

[B53] Potenza N. Vecchione C. Notte A. De Rienzo A. Rosica A. Bauer L. (2005). Replacement of K-Ras with H-Ras supports normal embryonic development despite inducing cardiovascular pathology in adult mice. EMBO Rep. 6 (5), 432–437. 10.1038/sj.embor.7400397 15864294 PMC1299307

[B54] Prior I. A. Hancock J. F. (2012). Ras trafficking, localization and compartmentalized signalling. Semin. Cell Dev. Biol. 23 (2), 145–153. 10.1016/j.semcdb.2011.09.002 21924373 PMC3378476

[B55] Prior I. A. Lewis P. D. Mattos C. (2012). A comprehensive survey of Ras mutations in cancer. Cancer Res. 72 (10), 2457–2467. 10.1158/0008-5472.CAN-11-2612 22589270 PMC3354961

[B56] Punekar S. R. Velcheti V. Neel B. G. Wong K. K. (2022). The current state of the art and future trends in RAS-targeted cancer therapies. Nat. Rev. Clin. Oncol. 19, 637–655. 10.1038/s41571-022-00671-9 36028717 PMC9412785

[B57] Pylayeva-Gupta Y. Grabocka E. Bar-Sagi D. (2011). RAS oncogenes: Weaving a tumorigenic web. Nat. Rev. Cancer 11 (11), 761–774. 10.1038/nrc3106 21993244 PMC3632399

[B58] Ramirez C. Hauser A. D. Vucic E. A. Bar-Sagi D. (2019). Plasma membrane V-ATPase controls oncogenic RAS-induced macropinocytosis. Nature 576 (7787), 477–481. 10.1038/s41586-019-1831-x 31827278 PMC7048194

[B59] Raso E. (2020). Splice variants of RAS-translational significance. Cancer Metastasis Rev. 39 (4), 1039–1049. 10.1007/s10555-020-09920-8 32772213 PMC7680328

[B60] Riely G. J. Johnson M. L. Medina C. Rizvi N. A. Miller V. A. Kris M. G. (2011). A phase II trial of Salirasib in patients with lung adenocarcinomas with KRAS mutations. J. Thorac. Oncol. 6 (8), 1435–1437. 10.1097/JTO.0b013e318223c099 21847063

[B61] Salmon M. Paniagua G. Lechuga C. G. Fernandez-Garcia F. Zarzuela E. Alvarez-Diaz R. (2021). KRAS4A induces metastatic lung adenocarcinomas *in vivo* in the absence of the KRAS4B isoform. Proc. Natl. Acad. Sci. U. S. A. 118 (30), e2023112118. 10.1073/pnas.2023112118 34301865 PMC8325162

[B62] Shimizu K. Birnbaum D. Ruley M. A. Fasano O. Suard Y. Edlund L. (1983). Structure of the Ki-ras gene of the human lung carcinoma cell line Calu-1. Nature 304 (5926), 497–500. 10.1038/304497a0 6308465

[B63] Simanshu D. K. Nissley D. V. McCormick F. (2017). RAS proteins and their regulators in human disease. Cell 170 (1), 17–33. 10.1016/j.cell.2017.06.009 28666118 PMC5555610

[B64] Sperlich B. Kapoor S. Waldmann H. Winter R. Weise K. (2016). Regulation of K-Ras4B membrane binding by calmodulin. Biophys. J. 111 (1), 113–122. 10.1016/j.bpj.2016.05.042 27410739 PMC4945620

[B65] Spiegelman N. A. Zhang X. Jing H. Cao J. Kotliar I. B. Aramsangtienchai P. (2019). SIRT2 and lysine fatty acylation regulate the activity of RalB and cell migration. ACS Chem. Biol. 14 (9), 2014–2023. 10.1021/acschembio.9b00492 31433161 PMC6893912

[B66] Stephen A. G. Esposito D. Bagni R. K. McCormick F. (2014). Dragging ras back in the ring. Cancer Cell 25 (3), 272–281. 10.1016/j.ccr.2014.02.017 24651010

[B67] To M. D. Wong C. E. Karnezis A. N. Del Rosario R. Di Lauro R. Balmain A. (2008). Kras regulatory elements and exon 4A determine mutation specificity in lung cancer. Nat. Genet. 40 (10), 1240–1244. 10.1038/ng.211 18758463 PMC2654781

[B68] Tsai F. D. Lopes M. S. Zhou M. Court H. Ponce O. Fiordalisi J. J. (2015). K-Ras4A splice variant is widely expressed in cancer and uses a hybrid membrane-targeting motif. Proc. Natl. Acad. Sci. U. S. A. 112 (3), 779–784. 10.1073/pnas.1412811112 25561545 PMC4311840

[B69] Vigil D. Cherfils J. Rossman K. L. Der C. J. (2010). Ras superfamily GEFs and GAPs: Validated and tractable targets for cancer therapy? Nat. Rev. Cancer 10 (12), 842–857. 10.1038/nrc2960 21102635 PMC3124093

[B70] Voice J. K. Klemke R. L. Le A. Jackson J. H. (1999). Four human ras homologs differ in their abilities to activate Raf-1, induce transformation, and stimulate cell motility. J. Biol. Chem. 274 (24), 17164–17170. 10.1074/jbc.274.24.17164 10358073

[B71] Wang M. T. Holderfield M. Galeas J. Delrosario R. To M. D. Balmain A. (2015). K-ras promotes tumorigenicity through suppression of non-canonical Wnt signaling. Cell 163 (5), 1237–1251. 10.1016/j.cell.2015.10.041 26590425

[B72] Wang Y. You M. Wang Y. (2001). Alternative splicing of the K-ras gene in mouse tissues and cell lines. Exp. Lung Res. 27 (3), 255–267. 10.1080/019021401300054028 11293328

[B73] Warburg O. (1956). On respiratory impairment in cancer cells. Science 124 (3215), 269–270. 10.1126/science.124.3215.269 13351639

[B74] Waters A. M. Ozkan-Dagliyan I. Vaseva A. V. Fer N. Strathern L. A. Hobbs G. A. (2017). Evaluation of the selectivity and sensitivity of isoform- and mutation-specific RAS antibodies. Sci. Signal. 10 (498), eaao3332. 10.1126/scisignal.aao3332 28951536 PMC5812265

[B75] Willumsen B. M. Christensen A. Hubbert N. L. Papageorge A. G. Lowy D. R. (1984). The p21 ras C-terminus is required for transformation and membrane association. Nature 310 (5978), 583–586. 10.1038/310583a0 6087162

[B76] Wright L. P. Philips M. R. (2006). Thematic review series: Lipid posttranslational modifications. CAAX modification and membrane targeting of ras. J. Lipid Res. 47 (5), 883–891. 10.1194/jlr.R600004-JLR200 16543601

[B77] Yang I. S. Kim S. (2018). Isoform specific gene expression analysis of KRAS in the prognosis of lung adenocarcinoma patients. BMC Bioinforma. 19 (1), 40. 10.1186/s12859-018-2011-y PMC6389035 29504894

[B78] Ying H. Kimmelman A. C. Lyssiotis C. A. Hua S. Chu G. C. Fletcher-Sananikone E. (2012). Oncogenic Kras maintains pancreatic tumors through regulation of anabolic glucose metabolism. Cell 149 (3), 656–670. 10.1016/j.cell.2012.01.058 22541435 PMC3472002

[B79] Yun J. Rago C. Cheong I. Pagliarini R. Angenendt P. Rajagopalan H. (2009). Glucose deprivation contributes to the development of KRAS pathway mutations in tumor cells. Science 325 (5947), 1555–1559. 10.1126/science.1174229 19661383 PMC2820374

[B80] Zhang X. Cao J. Miller S. P. Jing H. Lin H. (2018). Comparative nucleotide-dependent interactome analysis reveals shared and differential properties of KRas4a and KRas4b. ACS Cent. Sci. 4 (1), 71–80. 10.1021/acscentsci.7b00440 29392178 PMC5785771

[B81] Zhao H. Liu P. Zhang R. Wu M. Li D. Zhao X. (2015). Roles of palmitoylation and the KIKK membrane-targeting motif in leukemogenesis by oncogenic KRAS4A. J. Hematol. Oncol. 8, 132. 10.1186/s13045-015-0226-1 26715448 PMC4696201

